# Photo-sensitive degron variants for tuning protein stability by light

**DOI:** 10.1186/s12918-014-0128-9

**Published:** 2014-11-18

**Authors:** Svetlana Usherenko, Hilke Stibbe, Massimiliano Muscò, Lars-Oliver Essen, Ekaterina A Kostina, Christof Taxis

**Affiliations:** Department of Biology/Genetics, Philipps-Universität Marburg, Karl-von-Frisch-Strasse 8, 35043 Marburg, Germany; Department of Mathematics and Computer Science/Numerical Optimization, Universität Marburg, Hans-Meerwein-Straße 6, 35032 Marburg, Germany; Department of Chemistry/Biomedical Research Center, Philipps-Universität Marburg, Hans-Meerwein-Strasse 4, 35032 Marburg, Germany

**Keywords:** Optogenetics, LOV2 domain, Degron, Proteolysis, Light, Proteasome, Synthetic biology

## Abstract

**Background:**

Regulated proteolysis by the proteasome is one of the fundamental mechanisms used in eukaryotic cells to control cellular behavior. Efficient tools to regulate protein stability offer synthetic influence on molecular level on a selected biological process. Optogenetic control of protein stability has been achieved with the photo-sensitive degron (psd) module. This engineered tool consists of the photoreceptor domain light oxygen voltage 2 (LOV2) from *Arabidopsis thaliana* phototropin1 fused to a sequence that induces direct proteasomal degradation, which was derived from the carboxy-terminal degron of murine ornithine decarboxylase. The abundance of target proteins tagged with the psd module can be regulated by blue light if the degradation tag is exposed to the cytoplasm or the nucleus.

**Results:**

We used the model organism *Saccharomyces cerevisiae* to generate psd module variants with increased and decreased stabilities in darkness or when exposed to blue light using site-specific and random mutagenesis. The variants were characterized as fusions to fluorescent reporter proteins and showed half-lives between 6 and 75 minutes in cells exposed to blue light and 14 to 187 minutes in darkness. In blue light, ten variants showed accelerated degradation and four variants increased stability compared to the original psd module. Measuring the dark/light ratio of selected constructs in yeast cells showed that two variants were obtained with ratios twice as high as in the wild type psd module. *In silico* modeling of photoreceptor variant characteristics suggested that for most cases alterations in behavior were induced by changes in the light-response of the LOV2 domain.

**Conclusions:**

In total, the mutational analysis resulted in psd module variants, which provide tuning of protein stability over a broad range by blue light. Two variants showed characteristics that are profoundly improved compared to the original construct. The modular usage of the LOV2 domain in optogenetic tools allows the usage of the mutants in the context of other applications in synthetic and systems biology as well.

**Electronic supplementary material:**

The online version of this article (doi:10.1186/s12918-014-0128-9) contains supplementary material, which is available to authorized users.

## Background

In the last decade, light has been picked up as signal to control cellular behavior taking advantage of natural or engineered photoreceptors that regulate the activity of diverse output domains. This research field is called optogenetics and has recently attracted attention due to the unmatched characteristics of light as signaling entity, mainly the application of light with high temporal and spatial control [1]. The increasing importance of optogenetics for biomedical research is reflected in the development of diverse tools like microbial rhodopsins to control the nervous system of higher eukaryotes, usage of phytochromes and cryptochromes to control transcription in prokaryotes and eukaryotes, light oxygen voltage 2 (LOV2) domain-based control of a small GTPase or a formin, and the usage of the LOV2 domain or phytochromes to control protein localization [2-8]. Especially the LOV2 domain, which originates from plant phototropins, has been selected repeatedly as a tool to establish light-control of protein activity [9-12]. The LOV2 domain consists of a core domain with a Per-ARNT-Sim fold, which binds flavin mononucleotide (FMN) noncovalently as cofactor. An amphipathic helix named the Jα helix follows C-terminally to the core domain after a short loop consisting of a few residues [13,14]. The Jα helix is additionally linked to the core by a series of noncovalent interactions involving hydrophobic as well as polar amino acids [13,15]. Blue-light exposure of a LOV2 domain induces excitation of the FMN cofactor, which leads to the formation of a covalent cysteinyl-flavin C4a adduct that results in a conformational change of the core domain, detachment of the Jα helix from the core and subsequent unfolding of the helix [16]. This light-induced reaction takes place in the *Arabidopsis thaliana* LOV2 domain on a very short time-scale. The time constants have been measured to be 2 μs for photon absorption and adduct formation, 1 ms for the subsequent unfolding of the Jα helix, and about 70 s for the reversion to the dark state. The latter conversion includes transition of FMN to the ground state and refolding of the Jα helix [17]. Dark state reversion varies widely between different LOV domains with timescales spanning from seconds to days [18], which has been in the focus of many studies aiming to uncover the structural features responsible for the differences in reversion kinetics. These efforts led to the identification of several residues in the core domain close to the FMN cofactor that influence reversion to the dark state [19-27]. In addition, the Jα helix has been recognized as a region, which is important for the light-reaction of LOV2 domains. Using the *Avena sativa* LOV2 domain, it was shown that amino acid exchanges within the helix alter the signaling characteristics and affect both, the behavior in darkness as well as the behavior upon blue-light illumination [28,29]. Pseudo-lit-state mutants that show constant Jα helix undocking have been obtained by mutating residues in the Jα helix and residues near the N-terminus of the LOV2 domain [15,30]. The benefit of these efforts was information how LOV2 domains sense light and respond to it as well as the ability to change the light-response of optogenetic tools based on this widely used domain [24,28]. Recently, the structure of the *A. thaliana* phototropin1 LOV2 domain has been published. Strikingly, the Jα helix seems to comprise more residues compared to the homologous LOV2 domain from *Avena sativa* [14].

Most mutant variants of the LOV2 domain that increase the dissociation of the Jα helix from the core domain have been obtained by strategies, which favor the recovery of pseudo-lit state mutants [15,29,30]. However, it is desirable to obtain mutants that react profoundly to low amounts of light, but have in darkness a tight association of the helix to the core domain. Such mutants might be less important for constructs inducing site-specific activation of effector proteins on a short time-scale like photo-activatable Rac or photo-activatable formin [2,8], but are certainly interesting for applications that require long exposure to blue light as it is the case in optogenetic control of gene expression or of protein stability [3,31-35] to minimize the possibility of adverse effects due to long blue-light expositions of cells.

Control over protein stability has been achieved with the photo-sensitive degron (psd) module. It allows destabilization of selected proteins upon blue-light illumination by fusing it to the carboxy-terminal end of the target. It is composed of the *Arabidopsis thaliana* phototropin1 LOV2 domain and a synthetic degradation sequence (degron) called cODC1 that has been derived from the carboxy-terminal degron of murine ornithine decarboxylase (ODC). The ODC degron consists of a stretch of 37 amino acids without secondary structure and a cysteine-alanine motif, which is essential for activity of the degron. The cysteine has been shown to be required for proteasomal association, and has to be 19 amino acids away from the carboxy terminus of the protein [36,37]. In the psd module, a 23 amino acid long synthetic variant of the ODC degron has been fused directly to the end of the LOV2 Jα helix. This engineered degron is inactive in darkness due to the helical fold upstream of the cysteine-alanine motif and is activated by Jα helix unfolding. The psd module has been used to regulate diverse cellular functions in *Saccharomyces cerevisiae* by light [35]; a similar construct has been developed recently for usage in higher eukaryotes [31]. Here, we report the generation of photo-sensitive degron module variants that are useful to tune protein stability over a broad range with blue light. We characterized the impact of mutations in the LOV2 domain described in the literature as well as mutations obtained by random mutagenesis. *In vivo* and *in silico* characterization of the variants demonstrated that we obtained psd modules with increased degradation rate and higher dark/light ratio. Thus, we obtained psd modules that differ profoundly in their dynamic characteristics and will facilitate light-driven depletion of selected target proteins.

## Results and discussion

### Variants of the psd module with changed light-reactivity

**Figure 1:**
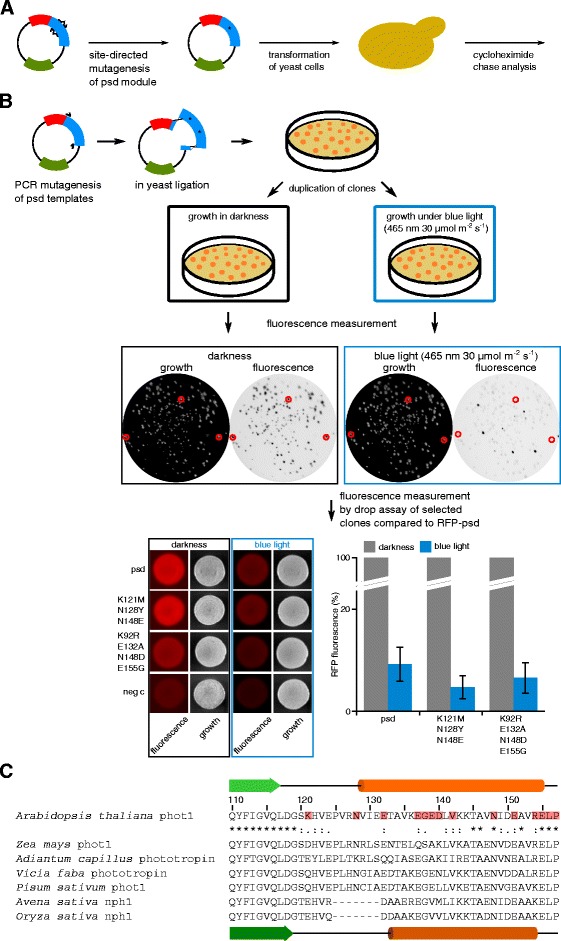
**Strategy to obtain psd module variants. A)** Site-directed mutagenesis was used to generate mutants within the Jα helix. After verification of the construct, yeast cells were transformed with the novel plasmid and subjected to cycloheximide chase analysis. **B)** Screening procedure to obtain mutants with altered psd module-characteristics. The plasmids pCT337 and pDS91 were used as template during random mutagenesis of the LOV2-cODC1 construct. The PCR products were combined with linearized vector (pDS90) and ligated in yeast by homologous recombination. The yeast clones were grown in darkness on selective solid medium, duplicated and either exposed to blue light or kept in darkness. The RFP fluorescence intensity of each clone was obtained for each condition. Clones with a promising dark/light ratio were selected for patch assays in comparison with the psd module (ESM356-1 + pDS90) as well as a negative control (neg c; ESM356-1 + pRS315) after growth in darkness and under blue light. At least four independent measurements were performed for each clone (lower right graph, error: s.e.m.). **C)** Sequence alignment of Jα helix-forming residues of LOV2 domains from different phototropins: *A. thaliana* phot1 (BAD94575.1 residues 569-616), *Z. mays* phototropin-1 (NP_001147477.1; 476-523), *A. capillus* phototropin (BAA95669.1|:661-708), *V. faba* phototropin (BAC23098.1; 540-587), *P. sativum* PsPK4 (AAB41023.2; 542-589), *A. sativa* nph1 (AAC05083.1; 507-547), and *O. sativa* nph1 (ABG21841.1; 202-242) were created with the software ClustalX. The secondary structure of *A. thaliana* LOV2 is shown on top (green arrow: β strand, orange tube: α helix) and the one of *A. sativa* (dark green arrow: β strand, dark orange tube: α helix) below the alignment. The numbering follows the sequence of the psd module. The grade of conservation of an amino acid is indicated using the ClustalX convention. Residues, which are mutated in psd module variants, are indicated by a red box.

We aimed to obtain psd module variants with increased destabilizing activity at a blue light illumination intensity that does not influence the growth rate of yeast cells (Additional file 1: Figure S1A and [35]). Even, *YAP1* mutant cells that have been reported to be very light-sensitive [38], are still able to grow under these conditions, although slower than wild type cells (Additional file 1: Figure S1B). Different approaches were taken to obtain improved psd module variants: Firstly, mutations that are known to change the characteristics of homologous LOV domains were selected and introduced into the psd module (Figure 1A). Domain swapping between two closely related LOV2 domains has demonstrated that the Jα helix region caused differences in dark-state recovery [25]. Thus, we selected mostly mutants within the Jα helix. This approach yielded the mutants V19I, G138A, E139N, and N148E, which correspond to the *As*LOV2 mutants V416I, G528A, V529N, and N538E [19,28,29]. Please note that the numbering of the psd module mutants starts at the methionine of the *At*LOV2 domain in the psd module; Additional file 1: Table S1 gives an overview of the mutants and the numbering of the residues in the full length *A. thaliana* phototropin1.

Secondly, a random mutagenesis was performed using the psd module and the N148E variant as templates to create a library of plasmids by in yeast ligation. The N148E mutant was chosen in addition to the wild type psd construct because the corresponding mutant in *As*LOV2 (N538E) was reported to have reduced Jα helix unfolding in darkness [28], which might be helpful in obtaining a psd module variant with increased dark/light ratio. In these constructs, the red fluorescence protein (RFP) was fused to the amino-terminal end of the psd module as reporter domain. The plasmid library was screened for yeast colonies that showed robust RFP fluorescence after growth in darkness and loss of fluorescence when exposed to blue light. This procedure yielded the mutants V142G, K92R E132A N148D E155G, and K121M N128Y N148E (Figure 1B).

Finally, we created variants by site-directed mutagenesis combining single mutations or changing residues in the Jα helix. Our aim was to destabilize the psd module in blue light without affecting its stability in darkness. Our main criteria was to combine mutations that produced interesting results during the first round of characterization and to generate mutants within the Jα helix that disfavor an α-helical conformation. For the latter attempt, experimentally obtained helix propensity values were used [39] to select amino acid exchanges that result in altered helix propensity, but do not disturb other characteristics profoundly. We made an alignment of the Jα helix region from *A. thaliana* LOV2 with homologous proteins to indicate all mutagenized residues in relation to conserved features. Moreover, the alignment demonstrates that in phototropins the loop connecting the core domain with the Jα helix is quite variable in sequence (Figure 1C). Remarkably, this loop of about 11 amino acids (D^568^GSKHVEPVRN^578^) is not fully resolved in the structure of the *At*LOV2 domain [14].

**Figure 2:**
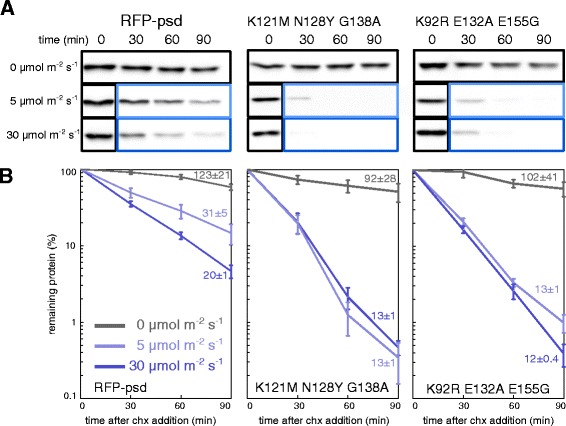
**Quantification of psd module variant behavior. A)** Yeast cells expressing P_*ADH1*_ *-RFP-psd* (plasmid based) or the variants K121M N128Y G138A and K92R E132A E155G were grown in liquid medium in darkness. After removal of the first sample (t=0 hours), cycloheximide (chx) was added to stop protein synthesis; cells were kept in the dark (black box) or exposed to blue light (LED lamp, 465 nm, light blue box: 5 μmol m^-2^ s^-1^, dark blue box: 30 μmol m^-2^ s^-1^) for the rest of the experiment. Equal amounts of sample were collected at the indicated time points and subjected to alkaline lysis and western blotting. **B)** Quantification of the immunoblots shown in **A**. Curves are the means obtained from at least four independent measurements (error bars: s.e.m.). The half-lives (±standard error) that are indicated next to each curve were obtained by fitting the data to an exponential decay using the software Origin 7.

So far, all the psd module variants contained the synthetic cODC1 sequence, which might differ in its degron activity from the native murine ODC degron. Thus, we created a psd module variant with the last 23 amino acids of the carboxy terminus of murine ornithine decarboxylase (named deg_ODC_) fused to the *At*LOV2 domain instead of the 23 amino acids long synthetic cODC1 sequence used in the psd module. As in the original construct, the unfolding of the LOV2 Jα helix is expected to complete the necessary number of unfolded amino acids required to induce proteasomal degradation. In addition, we tried to increase proteasomal association of the psd module upon activation by blue light. To do so, we multiplied the cysteine-alanine motif within the cODC1 sequence to have a double (CACA) or triple (CACACA) motif. In total, we analyzed 24 variants of the psd module for their stability in darkness or under blue light in yeast cells using cycloheximide chase analysis (Figure 2A and Additional file 1: Figure S2A,B). In this assay, the translational inhibitor cycloheximide was used to stop protein synthesis, which allowed us to follow the stability of a selected protein over time by western blotting. We found that the psd module variants were present in yeast cells in good amounts and showed robust light-response, which indicates that the variants were able to fold properly.

### Characterization of psd module variants

We quantified the western blots for each variant and fitted the curves to a first order exponential decay to obtain the half-lives in darkness and blue light (Figure 2B, Table 1, and Additional file 1: Figure S3). The observed half-lives in light ranged from 6.4 ± 0.2 min (R154G E155S) to 75 ± 14 min (G138A N148E) compared to 20 ± 1 min measured for the wild type psd module. In total, 10 of the variants showed a significant reduction in stability and four constructs were stabilized compared to the original construct. In darkness, we obtained values from 14 ± 2 min (R154G E155S) to 187 ± 45 min (ΔL156 ΔP157), while we found a half-life of 123 ± 21 min in the wild type psd construct. We measured several psd module variants at very low amounts of blue light (5 μmol m^−2^ s^−1^) and observed qualitatively similar results (Figure 2A,B, Additional file 1: Figure S2C and S3). For most constructs, the half-lives were somewhat prolonged and ranged from 24 ± 1 to 40 ± 3 min at 5 μmol m^−2^ s^−1^ (31 ± 5 min in psd) compared to 10 to 22 min at 30 μmol m^−2^ s^−1^ in these constructs (20 ± 1 min in psd). However, we identified five mutants that showed almost no difference between the two illumination conditions. We measured half-lives between 9.2 ± 0.5 and 13 ± 1 min in the K121M N128Y, K92R E132A E155G, G138A V142A R154G E155S, and K121M N128Y G138A variants at 5 μmol m^−2^ s^−1^ and 8.5 ± 0.5 and 13 ± 1 min in the same mutants at 30 μmol m^−2^ s^−1^. In the V19I variant, we observed have half-lives of 17 min at both illumination conditions. This mutation corresponds to the *A. sativa* LOV2 mutation V416I, which has been shown to prolong the dark state recovery time [19]. Thus, the high degradation rate at 5 μmol m^−2^ s^−1^ in the variants K121M N128Y, K92R E132A E155G, G138A V142A R154G E155S, and K121M N128Y G138A might be caused by prolonged dark-state recovery, either induced by changes of the photocycle or extended Jα helix refolding time. Remarkably, the psd module variant K121M N128Y has a half-life more than 3 times lower than the original construct at 5 μmol m^−2^ s^−1^ (Additional file 1: Figure S3). The four variants K121M N128Y, K92R E132A E155G, G138A V142A R154G E155S, and K121M N128Y G138A were obtained in the second round of psd module variant generation, in which stabilizing and destabilizing mutations were mixed in an attempt to optimize the constructs.

**Table 1:** **Characteristics of psd module variants**

**Name**	**Half-life in darkness (min)**	**Half-life in 30 μmol m** ^**−2**^ **s** ^**−1**^ **blue light (min)**	**Dark/light ratio of fluorescence measurements**
Wild type psd module	123 ± 21	20 ± 1	10.8 ± 0.5
V19I	132 ± 50	17 ± 2	n.d.
K92R E132A E155G	102 ± 41	12 ± 0.4	21.7 ± 2.5*
K92R E132A N148D E155G	103 ± 26	10.5 ± 0.3	12.2 ± 1.1
K92R E132A E139N N148D E155G	66 ± 10	9.8 ± 0.4	16.3 ± 1.2*
K121M N128Y	44 ± 8	8.5 ± 0.3	14.3 ± 1.4*
K121M N128Y G138A	92 ± 28	13 ± 1	21.7 ± 4*
K121M N128Y N148E	87 ± 18	17 ± 2	9.5 ± 0.5
E132D E139K	89 ± 25	17 ± 3	n.d.
E137D	165 ± 43	24 ± 4	n.d.
E137D E151D	79 ± 25	21 ± 3	n.d.
G138A	147 ± 52	22 ± 1	n.d.
G138A V142A R154G E155S	42 ± 10	10.5 ± 0.8	14.4 ± 1.4*
G138A N148E	151 ± 53	75 ± 14	n.d.
G138A R154G E155S	27 ± 4	7 ± 0.3	n.d.
E139N	89 ± 22	11 ± 0.5	10.3 ± 0.4
V142G	31 ± 4	19 ± 3	n.d.
N148E	168 ± 53	39 ± 8	n.d.
N148E R154G E155S	91 ± 35	13 ± 1	13.5 ± 0.7*
E151D	103 ± 26	28 ± 6	n.d.
R154G E155S	14 ± 2	6.4 ± 0.2	n.d.
ΔL156 ΔP157	187 ± 45	52 ± 9	n.d.
deg_ODC_	86 ± 21	20 ± 3	n.d.
CACA	87 ± 21	20 ± 3	n.d.
CACACA	66 ± 13	16 ± 1	n.d.

The half-lives were obtained by cycloheximide chase analysis and the ratio by fluorescence intensity measurements of cells kept in darkness or exposed to blue light (30 μmol m^−2^ s^−1^). The asterisk (*) marks dark/light ratios that are considered to be statistically different from the ratio of psd (P <0.05).

**Figure 3:**
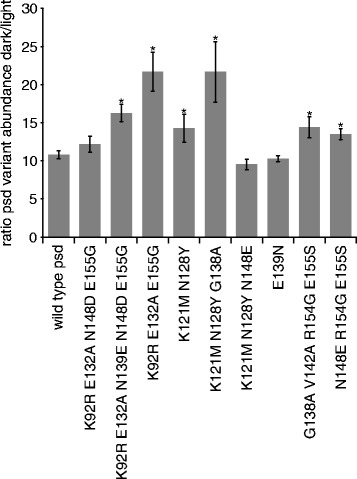
**Dark/light ratio of psd module variant abundance.** The protein levels of psd module variants were measured in yeast cells grown in darkness or exposed to blue light (LED lamp, 465 nm, 30 μmol m^-2^ s^-1^) for 5.5 hours by fluorimeter measurements. Plasmid encoded constructs were used for the *in vivo* measurements; all variants were derived from the psd module construct P_*ADH1*_ *-RFP-psd*. At least six independent measurements were performed for each construct. Error bars: s.e.m.; mean values with a statistically significant difference (P<0.05) to the mean value of the wild type psd module are indicated by an asterisk.

Next, the *in vivo* ratio of the abundance of the psd module variants in darkness and exposed to blue light was measured. We selected psd module variants with short half-life under blue light and at least moderate stability in darkness. The highest ratios were found in two mutants, K92R E132A E155G and K121M N128Y G138A. For both variants, the ratio was more than two times higher than in the wild type psd module. In the four variants K92R E132A N139E N148D E155G, K121M N128Y, G138A V142A R154G E155S, and N148E R154G E155S, we observed a moderate increase (about 1.4 fold) in the dark/light ratio (Figure 3, Table 1). Interestingly, all four variants with decreased stability at low illumination intensity (K92R E132A E155G, K121M N128Y, G138A V142A R154G E155S, and K121M N128Y G138A) show also an increase in dark/light ratio. The *in vivo* measurements highlighted the variants K92R E132A E155G and K121M N128Y G138A, which showed the highest dark/light switching factor and a faster degradation rate after blue-light illumination than the original construct (Figure 2, Figure 3, and Table 1). These two variants can be expected to be highly useful for *in vivo* manipulation of protein abundance by light.

The variants with short half-life under blue light showed increased turnover in darkness as well. We measured the impact of the ODC-like degron on protein stability in the variants K92R E132A E155G, K121M N128Y, K121M N128Y G138A, and G138A V142A R154G E155S by mutating the essential cysteine (C160) in the cODC1 degron. This resulted in profound stabilization in all variants under blue light (Additional file 1: Figure S4) and in darkness (data not shown), especially the K121M N128Y G138A variant showed almost complete stabilization. This indicates that the stability in the tested psd module variants depends mostly on cODC1 degron presentation.

**Figure 4:**
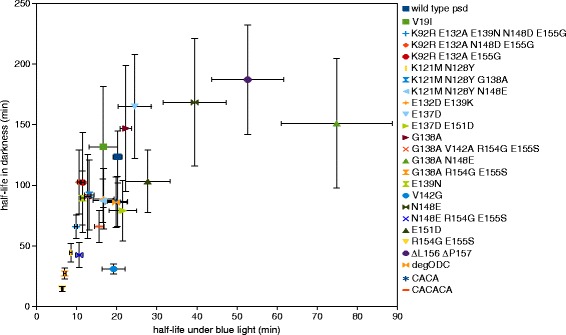
**Overview of the characteristics of psd module variants.** The half-life in darkness was plotted against the blue-light value for each construct (error bars: standard error).

Most of the psd module variants that we characterized followed a simple relation: the lower the *in vivo* stability in darkness, the lower the half-life in cells exposed to blue light (Figure 4). Notably, several psd module variants (G138A, E137D, V19I, N148E, ΔL156 ΔP157, G138A N148E) have similar half-lives in darkness (132 to 187 min), but different stability under blue light (17 to 75 min). This might indicate a very low rate of degron presentation in darkness in these variants, which results in a similar protein turnover rate.

The mutant with the shortest half-life (R154G E155S) does not seem to be particular useful in practical terms due to the very low half-life in darkness. The average half-life of all yeast proteins is 43 min [40], which means that most yeast proteins would be destabilized already in darkness if tagged with this psd module variant. Also attempts to stabilize this mutant (G138A R154G E155S and G138A V142A R154G E155S) were only partially successful in darkness (27 ± 4 min and 42 ± 10 min), whereas they retained their short half-life in blue light (7 ± 0.3 min and 10.5 ± 0.8 min). The triple mutant N148E R154G E155S is affected in darkness and blue light (91 ± 35 min; 13 ± 1 min) and has characteristics that are shifted towards the psd module. This series of variants demonstrates that it is possible to tune psd module stability to desired values with appropriate combinations of destabilizing and stabilizing mutations. Similar strategies are feasible for other optogenetic tools using the LOV2 domain as well.

The mutant K121M N128Y, which was derived from a mutant (K121M N128Y N148E) that was picked-up by random mutagenesis, showed a pronounced impact on the stability of the psd module in darkness (half-life 44 ± 8 min) and under blue light (8.5 ± 0.3 min). Interestingly, the K121M N128Y mutations map to the loop that connects the Jα helix with the LOV2 core domain. This region is very variable among plant LOV2 domains, demonstrated by lack of residue conservation (Figure 1C). Based on statistical coupling analysis, it has been proposed that this loop is a functional sector in the LOV domain family that may induce phenotypic variation [41]. The T517V loop mutation in *As*LOV2 showed an unaltered photocycle but increased conformational change upon illumination [42]. It may be of interest to study the impact of mutations in this loop in plant LOV2 domains in detail.

Mutations corresponding to G138A and N148E have been found to increase the dynamic range of the *Avena sativa* LOV2 domain by stabilizing the docking of the Jα helix to the core [28]. We found qualitatively similar effects in the psd module containing the *A. thaliana* LOV2 domain (Table 1). Especially, the G138A mutation in *At*LOV2 seems to reduce unfolding of the Jα helix in darkness, as has been observed for *As*LOV2 [28]. Interestingly, two mutants with very pronounced impact on psd module characteristics had both amino acid exchanges at the C-terminal border of the Jα helix. We found strong destabilization for the R154G E155S variant (darkness: 14 ± 2 min; light: 6.4 ± 0.2 min; wild type darkness: 123 ± 21 min; light: 20 ± 1 min) and pronounced stabilization for the ΔL156 ΔP157 mutant (darkness: 187 ± 45 min; light: 52 ± 9 min). This indicates sensitivity of this region towards changes in the amino acid composition. A possible explanation might be that interactions between these residues and amino acids of the core domain stabilize the Jα helix in the docked conformation. The crystal structure of *As*LOV2 [13] shows that the side chain of L546 is about 4 to 5 Å away from Y508 and F429, which is a distance sufficient for mutual interaction. Strickland and colleagues reported that the *As*LOV2 Y508K mutant shows slightly increased undocking of the helix in the dark compared to the wild type [28], whereas an exchange (I428T) in the residue that precedes F429 results in a shortened photocycle lifetime and an increased conformational change [42].

We designed three mutants (E137D, E151D, and E137D E151D) that were expected to destabilize the Jα helix due to changes in helix propensity. We anticipated that this should impact the behavior of the psd module in darkness and in blue light. However, we did not find a pronounced effect on the half-life of the psd module under blue light (Table 1, Additional file 1: Figure S2 and S3). In accordance with this, we did not observe a correlation of helix propensity values with half-life in darkness or under blue light analyzing all psd module variants with mutations in the Jα helix (data not shown). This might indicate that electrostatic interactions of the residues between each other and with residues of the core domain are more important for the stability of the helix than the helix propensity values of the helix residues. This is in agreement with findings from analysis of *As*LOV2 mutants, which implied that electrostatic interactions are important for the photoresponse of the protein [20]. Site directed exchanges in the degron part of the psd module did not result in a dramatic change of the psd module behavior. The CACACA mutant showed a decrease in half-life to 16 ± 1 min, whereas the CACA and the deg_ODC_ variants were similar to the wild type psd module (20 ± 1 min), although all three mutants showed decreased stability in darkness (Table 1, Additional file 1: Figure S2 and S3).

### Simulation of psd module behavior

Previously, we used the computer-aided design software TinkerCell [43] to generate a model that simulated the behavior of the psd module [35]. Among other things, the simulation and analysis functions of TinkerCell can be used to predict the steady state levels of proteins within a model or simulate dynamic behavior of a system after an initial change of parameters. The model of the psd module includes a protein synthesis part (pp1) simulating the production of the photo-sensitive degron module protein (PSD), light-driven conversion of the LOV2 dark-state to the lit-state (k_hν_), reversion back to the dark state (k_dark_), light-independent conversion to the lit-state (k_leak_), endogenous protein degradation (k_degENDO_) of PSD, and light-induced protein degradation (k_degLOV_) of PSD. The differential equations of the model, which we used to simulate psd module behavior, are given in Additional file 1: Figure S5A. In summary, the model simulates the synthesis and degradation of PSD in yeast cells. Comparison of the simulated levels of PSD with *in vivo* measurements of RFP-psd abundance were in good accordance, the simulations recapitulated quite well the light-response, kinetics of protein depletion, and the difference between abundance in darkness and under blue light illumination [35].

To gain a better quantitative understanding of the novel psd module variants, the conversion constants k_dark_, k_leak_, k_degLOV_, and k_degENDO_ were derived from simulations matching the experimentally derived curves (Additional file 1: Figure S6 and Table S2). We assumed that all mutants have unchanged quantum yields, which was justified by the localization of the mutations that are far from the residues involved in FMN binding. However, we cannot formally exclude that for some mutants the quantum yield might be slightly altered. The experimental data obtained by the cycloheximide chases provided more data points than free parameters in all numerical computations. However the experimental data alone did not guarantee parameter estimates with tight error ranges. A priori information about biological-meaningful parameter ranges was available from the cycloheximide chase experiments (Additional file 1: Figure S3) and literature [17,29,35-37,44,45]. According to the Bayesian approach this information must be taken into account. Indeed, a priori information (see Additional file 1: Table S3) together with the experimental data resulted in parameter estimates presented in Additional file 1: Table S2 with tight error ranges. For the psd module itself, changes were only allowed in k_degLOV_ and k_degENDO_, whereas k_leak_ and k_dark_ were derived from literature [29,35]. Parameter estimation using the curves obtained at light fluxes of 0, 5 and 30 μmol m^−2^ s^−1^ resulted in values for k_degLOV_ and k_degENDO_ very close to previously obtained results [35]. We simulated the impact of increased k_degLOV_ and decreased k_dark_ in cycloheximide chases compared to simulations with the values obtained for the wild type psd module (Additional file 1: Figure S7A). An increase in k_degLOV_ led to slightly faster degradation in darkness and visibly accelerated decay at 5 and 30 μmol m^−2^ s^−1^ (Additional file 1: Figure S7B). This graph resembled the experimental data obtained for the variants K92R E132A N148D E155G or K92R E132A N148D E155G (Additional file 1: Figure S3). A decrease in k_dark_ resulted in a graph that had almost no difference between the two illumination conditions and moderate increase in degradation in darkness (Additional file 1: Figure S7C). This is similar to the data obtained from the mutants V19I, K121M N128Y, K121M N128Y G138A, K92R E132A E155G or G138A V142A R154G E155S (Additional file 1: Figure S3).

Simulations with the estimated psd module parameters showed that 67% of the molecules occupy the lit state at 30 μmol m^−2^ s^−1^ blue light, 26% at 5 μmol m^−2^ s^−1^ and 2.5% in darkness (data not shown). This indicated that at blue light fluxes, which did not affect growth of yeast cells (Additional file 1: Figure S1A and [35]), only a fraction of the molecules is destabilized due to degron activation. The conversion constants obtained from the parameter estimation suggested that for some of the psd module variants a higher lit state fraction might be reached. Examples are the psd module variants V19I, K92R E132A E155G, K121M N128Y, K121M N128Y G138A, and R154G E155S, in which low values for k_dark_ and k_leak_ were estimated (Additional file 1: Table S2).

**Figure 5:**
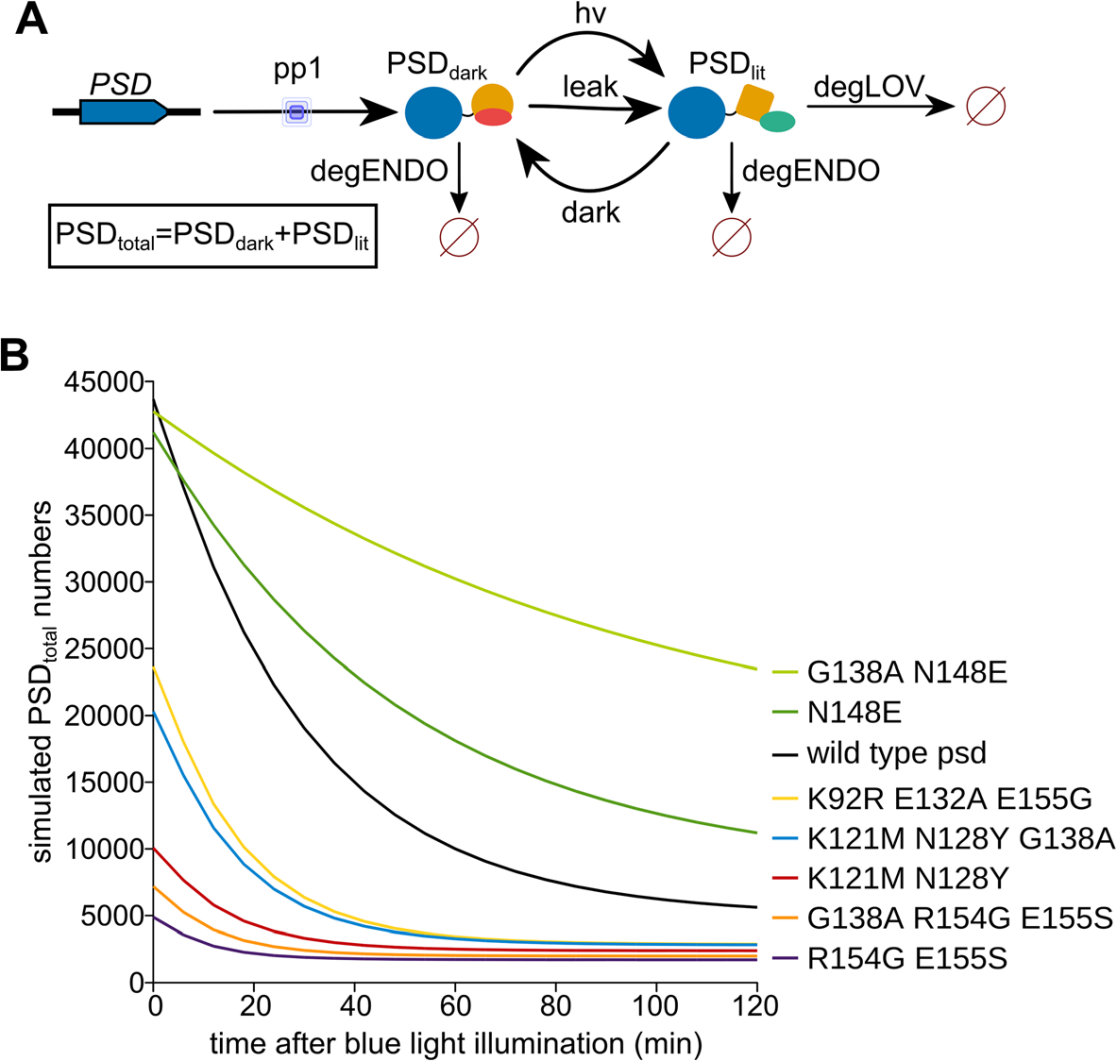
**Simulated reaction of psd module variants towards light. A)** Model used for the simulations, which contains the protein production module (pp1) as well as the degradation pathways. **B)** Kinetics of psd module variant depletion. For the simulations, darkness steady state numbers for PSD_dark_ and PSD_lit_ were used as starting values and an exposure to blue light (30 μmol m^-2^ s^-1^) was simulated between 0 min and 120 min.

We simulated the behavior of the psd module using the full model, which includes protein production and degradation (Figure 5A). We were interested to model the kinetics of psd module variant depletion at a light flux of 30 μmol m^−2^ s^−1^. The absolute change of PSD_total_ numbers was predicted to be higher for the psd module than for most variants with decreased half-life, but these variants reached lower molecule numbers (PSD_total_) much faster (between 40 and 60 min) upon illumination than wild type psd. As expected, slower depletion kinetics were predicted for variants with higher half-life (Figure 5B). In summary, the simulations demonstrated that the novel psd module variants can be used to influence target protein levels by light within a wide range. Most likely, variants with decreased half-life are of greater interest, but the variants give also the possibility to tag two or more target proteins with different psd modules and reach different target protein levels using only one signal.

## Conclusions

Our mutational approach to improve the psd module resulted not only in variants with decreased stability, but these variants showed a higher dark/light switching ratio as well. Our characterization of a set of psd module variants and subsequent *in silico* analysis complements previous studies using the light-regulated trp repressor LovTAP, the photo-controlled kinase YF1, and measurements in single LOV2 domains [21,22,28,42,46].

The novel variants of the photo-sensitive degron module show protein turnover rates that are similar to half-lives that have been measured with other degrons used for induced protein degradation. In yeast, the half-life of murine ODC degron fused to a mutant titin was determined to be about 6 min [47], similar to the psd module variants with shortest half-life. A faster degradation rate has been measured with an N-degron generated by the ubiquitin fusion technique; an exposed N-terminal arginine induced the degradation of a tester protein with a half-life of about 2 minutes [48]. For the temperature-sensitive degron, which uses an arginine as N-degron as well [49], similar half-lives can be expected in the best case. Another well-developed method is the plant auxin-inducible degron (AID) system for non-plant cells [50]. In yeast, half-lives of about 11 minutes have been determined with this system, measurements in mammalian cells resulted in half-lives between 9 and 18 minutes for different target proteins [51,52]. The novel psd module variants reported here showed half-lives that are comparable with the ones of established methods, which are commonly used to degrade target proteins using different signals for induction. The usage of the photo-sensitive degron requires C-terminal fusions to target proteins, which is imposed by the choosen degradation sequence and the mode of activation [35]. Target proteins have to expose the degron to the cytosol or nucleus to be available for regulation by the photo-sensitive degron module, a feature that is shared by all techniques that control protein stability by proteasomal degradation.

Light-mediated regulation has the huge advantage over temperature or chemicals that it can be precisely regulated in quantity, space and time, at least at the level of micro-organisms or cell cultures. A vertebrate-adapted variant of the photo-sensitive degron has been established in zebrafish embryos and mammalian cells, which demonstrates the generality of the method [31]. The continuous illumination that is required to induce depletion of a target protein asks for photo-sensitive degron variants that highly destabilize the target at low illumination strength. The variants we describe here improve the original construct profoundly in these two critical features.

## Methods

### Yeast strains and growth conditions

The *Saccharomyces cerevisiae* strains are derivatives of the S288C strain ESM356-1 (*MATa ura3-52 leu2Δ1 his3Δ200 trp1Δ63)* [53], except for the strains shown in Additional file 1: Figure S1, which are SK1-derivatives (YKS32 (*MAT***a**/*MAT****α****lys2/lys2 ura3/ura3 leu2/LEU2 ho*::*hisG/ho*::*LYS*) [54] and YMM30 (YKS32 *yap1Δ::kanMX/yap1Δ::kanMX*)). Standard preparations of media were used for growth on plates [55]. Yeast cells were transformed with plasmids by the lithium acetate method [56]. Low-fluorescence medium (100 ml salt stock [1% KH_2_PO_4_; 0.5% MgSO_4_; 0.1% NaCl; 0.1% CaCl_2_; 5% (NH_4_)_2_SO_4_], 0.1 ml trace element stock [50 mg H_3_BO_4_; 4 mg CuSO_4_; 10 mg KI; 20 mg FeCl_3_; 40 mg MnSO_4_; 20 mg Na_2_MoO_4_; 40 mg ZnSO_4_ in 100 ml ddH_2_O], 0.1 ml vitamin stock [0.2 mg biotin; 40 mg calcium pantothenate; 200 mg inositol; 40 mg niacin; 20 mg para-amino benzoic acid; 40 mg pyroxidine HCl; 40 mg thiamine HCl in 100 ml ddH_2_O], 100 ml 20% glucose, 2 g of the appropriate amino acid stock in 1 l ddH_2_O, sterile filtered) was used to grow yeast cells in liquid cultures in standard plastic cell culture flasks. Blue-light irradiation of yeast cells was performed using custom-build sets of light-emitting diodes (LEDs): high power LED stripes (18 LEDs, 465 nm; revoART, Borsdorf, Germany) or StrawHat LED clusters (6 clusters of 42 LEDs, 465 nm; revoART, Borsdorf, Germany); both sets were equipped with a dimmer to select an appropriate light-flux. For the experiments, light-fluxes of 30 or 5 μmol m^−2^ s^−1^ were used, the light-flux was checked before the experiment at the level of the yeast cells (distance yeast cells - LEDs: 10 cm) with an optometer (P2000, equipped with light-detector PD-9306-2, Gigahertz-Optik, Türkenfeld, Germany). Yeast cells with and without psd module variants showed no difference in growth in darkness or exposed to a blue-light flux of 30 μmol m^−2^ s^−1^ (Additional file 1: Figure S9).

### Plasmid construction, random mutagenesis and screening procedure

Plasmids were constructed by standard procedures [57], details and sequences of the used vectors are available on request; yeast plasmids are listed in Additional file 1: Table S4. The strategy to obtain psd module variants by random mutagenesis is shown in Figure 1B. The random mutagenesis was performed essentially as described [58] using the template plasmids pCT337 as well as pDS91 and the primers tagrfp_at_lov2_up (AGATATTGTGATTTACCATCTAAATTAGGTCATAAACTGCAGATGAGAAAGGGTATTGATCTAG) and ctermcODC_in_pCT323_downrev (GTGACATAACTAATTACATGACTCGAGTTATTGGAAGTACAAGTTTTCAGAAC). The yeast strain ESM356-1 was transformed with the PCR product together with the linearized plasmid pDS90 (PstI/XmaJI) and grown in darkness on selective solid medium. The clones were duplicated by replica plating, one plate was grown under blue light (465 nm, 30 μmol m^−2^ s^−1^), the other in darkness. The RFP fluorescence intensity of each clone was obtained with the fluorescence image analyzer Fujifilm LAS-4000 equipped with a 16-bit CCD-camera, white light (to image growth of yeast cells), green light-emitting LEDs (emission maximum 520 nm) and an emission filter set (575 nm-DF20) suitable for RFP observation. The blue light/darkness fluorescence ratio was calculated for each clone and the clones with smallest ratio were selected for further analysis. The selected clones were grown in patches together with the psd module (ESM356-1 + pDS90) in darkness as well as under blue light. After 24 hours, the RFP fluorescence was measured. Plasmids of clones that performed better than the psd module were rescued from yeast cells into *E. coli* by a standard procedure [57] and sequenced.

### Cycloheximide chase, immunoblotting, quantitative measurements, sequence alignments, and statistics

Yeast cells expressing P_*ADH1*_*-RFP-psd* (plasmid based) or variants thereof were grown in low fluorescence medium in the dark until logarithmic growth phase was reached. The first sample (t = 0 hours) was taken and the translation inhibitor cycloheximide (end concentration 200 μg/ml) was added to stop protein synthesis. Cells were kept in darkness or exposed to blue light for the rest of the experiment. Equal amounts of sample were collected at each time point and subjected to alkaline lysis and western blotting. Immunoblotting experiments were performed as described [58]. Quantification was done with the ImageJ program [59] using the gel analyzer tool. The background in the images was removed with the function < Process < Subtract Background (setting: rolling ball radius 50 pixels). Lifetimes were obtained using the fitting wizard (first order exponential decay) of the program Origin 7. These were converted into half-lives by multiplying them with the natural logarithm of two. At least four independent measurements were performed for each psd-module variant. The figures show representative results (immunoblotting) or mean results. Error bars show the standard error of the mean (s.e.m.). The flourimeter measurements to obtain dark/light ratios of the RFP-psd variant abundance were essentially done as described [58]. Briefly, logarithmically growing yeast cells (in liquid low fluorescence medium supplemented with 2% glucose) were exposed to blue light (30 μmol m^−2^ s^−1^) for 5.5 hours or kept in darkness for the same amount of time. Equal amounts of cells were taken from each condition, treated with sodium azide (10 mM final concentration), transferred to a black, flat-bottom 96-well microtiter plate (Greiner Bio-One, Germany) and the RFP fluorescence was measured with a microplate reader (Synergy Mx, BioTek, Bad Friedrichshall, Germany). Excitation conditions: 10 flashes of light (555 nm); fluorescence was observed at a wavelength of 585 nm with a gain of 130. Background fluorescence was obtained from yeast cells without construct and subtracted from the measurements of RFP-psd variant containing cells. The ratio was calculated by dividing the fluorescence intensity measured in darkness with the value obtained from cells exposed to blue light. At least six independent measurements were done for each construct. Statistical analysis (pairwise *t*-test) was done with the QuickCalcs online calculator (www.graphpad.com/quickcalcs/index.cfm). The software ClustalX with standard settings was used to perform sequence alignments [60]. The sequences were obtained from databases maintained by the NCBI (www.ncbi.nlm.nih.gov).

### Simulations of cycloheximide chase experiments

The model used to simulate the behavior of the psd module in cycloheximide chases (Additional file 1: Figure S7) was based on the model for the psd module [35] (shown also in Figure 5A) and was modified with the computer-aided design software TinkerCell [43]. The differential equations for cycloheximide chase simulation are shown in Additional file 1: Figure S5B. The protein production module was inactivated (k_pp1_translation_rate_ = 0 min^−1^) and a fixed number of molecules in the dark and the lit state was used as starting condition. We simulated psd module behavior in cycloheximide chases with the stochastic (exact) analysis function (30 timepoints; 90 minutes). Starting parameters were: k_degENDO_ = 0.0028 min^−1^, k_dark_ = 0.59 min^−1^, k_degLOV_ = 0.048 min^−1^, k_hν_ = 0 min^−1^ (darkness), k_hν_ = 0.2 min^−1^ (5 μmol m^−2^ s^−1^) or k_hν_ = 1.2 min^−1^ (30 μmol m^−2^ s^−1^), k_leak_ = 0.01513 min^−1^, k_pp1_mRNA_degradation_rate_ = 0.039 min^−1^. Initial values were: PSD_dark_ = 49000 molecules, PSD_lit_ = 1000 molecules, pp1_mRNA = 89 molecules, and psd = 3.3. The light conversion rate of k_hν_ = 0.0404 min^−1^ at a light flux of 1 μmol m^−2^ s^−1^ was calculated from the quantum yield of 0.26 for FMN [44] multiplied with the FMN cross section of 4.3*10^−17^ cm^2^ (at l_max_ = 450 nm) [45] and the number of photons (for a light flux of 1 μmol m^−2^ s^−1^, this is 6.023*10^13^ cm^−2^ s^−1^) [35].

Simulations of cycloheximide chase experiments were done for each variant to obtain starting values for parameter estimations (Additional file 1: Table S3). Please note that the half-life in darkness of the psd module and the variants is not solely reflected by k_degENDO_ in the model. Rather, unfolding of the Jα helix in darkness, which is reflected in the model by k_leak_, results in cODC1 exposure, which is contributing to the protein degradation in darkness. Furthermore, we use the term half-life throughout the publication to describe the *in vivo* stability of the psd module and its variants. This should make a clear distinction from the term lifetime, which is commonly used in photobiology to describe the photo-cycle of photoreceptors.

### Parameter estimation

The conversion rates k_dark_, k_leak_, k_degENDO_ and k_degLOV_ for the mutants and the wild type psd module were obtained by parameter estimation using the cycloheximide chase data. We estimated parameters by solving the following multiple experiment parameter estimation problem

1 $$ \underset{y^k,\ k=1,\dots, {N}_{\exp },p}{ \min }{\displaystyle \sum_k^{N_{exp}}}{\displaystyle \sum_j^{N_{meas}}}{\left(\frac{H_j^k-q\left({y}^k,\left({t}_j\right),,,p\right)}{\varSigma_j}\right)}^2+{\displaystyle \sum_{i=1}^{n_p}}{\left(\frac{p_i-{p}^{apriori}}{\varSigma}\right)}^2, $$

2 $$ s.t.\ \dot{y^k}=f\left({y}^k,{u}^k,p\right),\ t\in \left[0,{t}_f\right],\ {y}^k\left({t}_0\right)={y}_0^k,\ k=1,\dots, {N}_{exp}. $$

Here the dynamics of protein degradation in each experiment was modeled by a system of ordinary differential equations (ODE) (2), where ${y}^k\in {\mathbb{R}}^{n_y}$ denoted the states in the *k* -th experiment at time moment *t*, $p\in {\mathbb{R}}^{n_p}$ denoted parameters to be estimated and *u*_*k*_, *k* = 1, …, *N*_exp_ were controls in each of *N*_exp_ experiments. The cost functional (1) described the mismatch between the data ${H}_j^k$ and the measurement function. The cost functional also might include a term which characterizes an a priori information on the parameters (Additional file 1: Table S3). These information were obtained from cycloheximide chase simulations with TinkerCell and literature [17,29,35-37,44,45]. For each variant, parameters were chosen that approximated the experimental data. During parameter estimation, these approximations were then used as starting values. In the problem under consideration the states *y*(*t*) described the protein concentrations, *y*_1_(*t*) and *y*_2_(*t*) being PSD_dark_ and PSD_lit_ concentrations, respectively. The parameters *p*_1_, …, *p*_4_ were k_degENDO_, k_leak_, k_dark_and k_degLOV_. The controls *u*^1^ = 0, *u*^2^ = 0.2 and *u*^3^ = 1.2 described the light intensities in each of the three experiments. In each experiment, we had three measurements at *t*_1_ = 30, *t*_2_ = 60, *t*_3_ = 90. The measurement function was given by *q*(*y*(*t*), *p*) = *y*_1_(*t*) + *y*_2_(*t*).

The problem (1)-(2) was solved using the so-called Boundary Value Problem (BVP) Approach for parameter estimation in differential equations. The general idea of the BVP Approach is sketched here, further details of the BVP approach may be found in [61,62]. First a time domain was decomposed and the dynamical model was parametrized by multiple shooting: For an appropriate grid of *M* time points *Τ*_*j*_

$$ 0={T}_1<{T}_2<\dots <{T}_M={t}_f, $$

which covers the measurement interval [0, *t*_*f*_], the discrete trajectory ${s}_j^k:={y}^k\left({T}_j\right),\kern1em k=1,\dots, {N}_{\exp }$ was introduced as unknown variables in addition to the unknown parameters *p*. For a given guess for the extended variable vector $\left({s}_1^k,\dots, {s}_M^k,\kern1em k=1,\dots, {N}_{\exp },p\right)$ the solutions ${y}^k\left(t;{s}_j^k,p\right)$ of the *M* − 1 independent initial value problems

$$ {\dot{y}}^k=f\left({y}^k,p,{u}^k\right),{y}^k\left({T}_j\right)={s}_j^k,\kern1em t\in {I}_j:=\left[{T}_j,{T}_{j+1}\right[,\kern1em k=1,\dots, {N}_{\exp } $$

were calculated on each sub interval *I*_*j*_, which resulted in a (usually not continuous) ODE solution. The ODE solution at the measurement points ${y}^k\left({t}_i;{s}_{j(i)}^k,{u}^k,p\right)$ for *t*_*i*_ ∈ [*Τ*_*j*(*i*)_, *Τ*_*j*(*i*) + 1_[ were formally inserted into the cost functional (1). The continuity of the optimal ODE solution was ensured by the following constraints

$$ {y}^k\left({T}_{i+1};{s}_i^k,p\right)-{s}_{i+1}^k=0,\kern1em k=1,\dots, {N}_{\exp },\kern1em i=0,\dots, M-1, $$

which were included into the optimization problem.

As a result the parameter estimation problem in the ODE system was transformed into a finite dimensional optimization problem that could be written in the form

$$ min\frac{1}{2}{\left\Vert {F}_1\left.(x)\right\Vert \right.}_2^{2,}\kern1em s.t.\kern1em {F}_2(x)=0. $$

Note, that the equalities *F*_2_(*x*) = 0 included the matching conditions.

This problem was solved by a generalized Gauss-Newton method according to which the new iteration was computed by

$$ {x}^{k+1}={x}^k+{t}^k\varDelta {x}^k $$

where the increment *Δx*^*k*^ solves the quadratic problem

3 $$ \underset{\varDelta x\in {\varOmega}^k}{ \min}\frac{1}{2}{\left\Vert {F}_1\left.\left({x}^k\right),+\nabla {F}_1\left({x}^k\right)\varDelta x\right\Vert \right.}_2^{2,}{F}_2\left({x}^k\right)+\nabla {F}_2{\left({x}^k\right)}^T\varDelta x=0. $$

The linearized problem (3) showed special structures due to multiple experiments and multiple shooting approaches, which were efficiently exploited in a tailored linear algebra method for its solution. With this method the new parameters were computed.

A numerical analysis of the well-posedness of the problem and an assessment of the error of the resulting parameter estimates were performed at the solution p* of the problem (1)-(2). In particular the standard deviations of the parameter estimates were computed.

The parameter estimations were performed with fixed light conversion rates (k_hν_) at light fluxes of 0 and 30 μmol m^−2^ s^−1^ for the mutants and the wild type psd construct. For the wild type psd construct, we allowed a certain variation of k_hν_ (±10%) in case of the light flux of 5 μmol m^−2^ s^−1^ (k_hν_ = 0.379 ± 0.237 min^−1^ instead of 0.2 min^−1^). This did not change the values for the reaction constants k_dark_, k_leak_, k_degENDO_ and k_degLOV_ considerably, but lead to much better fitting of the simulated curves to the experimentally derived curves (data not shown). This might indicate that illumination conditions were not constant from one experiment to the other during the measurements of the wild type psd module at 5 μmol m^−2^ s^−1^. For the mutant psd variants, no adjustment of the light conversion rate k_hν_ at 5 μmol m^−2^ s^−1^ was necessary to obtain good fittings.

The half-life of the K121M N128Y variant exposed to blue light (465 nm, 30 μmol m^−2^ s^−1^) was used as starting parameter to estimate the k_degLOV_ conversion rate (0.0564 min^−1^ ± 10% for most fittings), because this mutant showed the fastest depletion rate of the variants that do not contain a mutation in a region that could influence cODC degron strength. The border was set to residue K143, 37 amino acids away from the carboxy-terminal end of the protein, assuming that only within this region changes might influence degron activity. Thus, the conversion constant k_degLOV_ was freely adapted, if amino acid exchanges occurred between K143 and the end of the protein.

The conversion rate for endogenous protein turnover (k_degENDO_) was kept fairly constant (0.0025 ± 10%) for all variants assuming that none of the mutations has a considerable impact on protein folding. This was justified by the observation that the variants were detected in good amounts by immunoblotting and showed robust light-reactivity. In addition, we measured the impact of the cODC1 part by mutating the critical cysteine. This resulted in strong stabilization in all tested cases (Additional file 1: Figure S4). Furthermore, we performed TinkerCell simulations with psd module values increasing only k_degENDO_. However, this did not reproduce the experimental data of the variants (data not shown).

Values for k_dark_ = 0.59 min^−1^ (70 sec dark recovery time) and k_leak_ = 0.01513 min^−1^ (2.5% of the molecules in lit state in darkness) [29,35] were used for the psd module and the variants deg_ODC_, CACA, and CACACA, in which only the cODC1 part was modified. For the other psd variants, the values for k_dark_ and k_leak_ were freely adapted, whereas k_degLOV_ and k_degENDO_ were kept within borders as described above. Furthermore, we restricted the values of all conversion rates to exclude solutions with negative parameters. The total amount of PSD (PSD_total_ = PSD_dark_ + PSD_lit_) was used to generate graphs.

### Simulation of the characteristics of psd module variants

The full model [35] (also shown in Figure 5A, differential equations Additional file 1: Figure S5A) including protein production and degradation was used to simulate the behavior of the psd variants over time. To do so, PSD_total_ numbers were simulated over a time period of 120 min after light exposure (30 μmol m^−2^ s^−1^). First, the molecule numbers for time point 0 were obtained from simulating steady state levels of PSD_dark_ and PSD_lit_ in darkness for each variant. The < steady state < get state function of TinkerCell was used with following parameters: k_dark_, k_leak_, k_degENDO_ and k_degLOV_ as shown in Additional file 1: Table S2 for each variant, k_pp1_translation rate_ =2 min^−1^, k_pp1_mRNA_degradation_rate_ = 0.039 min^−1^. Initial values were: pp1_mRNA = 89 molecules, psd = 3.3. The obtained values were used as initial values for PSD_dark_ and PSD_lit_ molecule number in the following simulations. There, the < simulation < stochastic (exact) function was used with following parameters: k_dark_, k_leak_, k_degENDO_ and k_degLOV_ as shown in Additional file 1: Table S2 for each variant, k_pp1_translation rate_ =2 m^−1^, k_pp1_mRNA_degradation_rate_ = 0.039 min^−1^. Other initial values: pp1_mRNA = 89 molecules, psd = 3.3.

### Availability of supporting data

The data sets supporting the results of this article are included within the article (and its additional file).

## Additional file

Additional file 1: Figure S1 Growth behavior of yeast cells in blue light compared to darkness. **Figure S2.** Mutational analysis of psd module stability. **Figure S3.** Quantification of psd module variant behavior. **Figure S4.** The impact of the cysteine within the cODC1 degron on psd module variant stability. **Figure S5.** Equations used for the *in silico* analysis of psd module variants. **Figure S6.** Parameter estimation to the experimental data of the psd module variants. **Figure S7.** Influence of increased k_degLOV_ and decreased k_dark_ on psd module behavior. **Figure S8.** Comparison of human parameter assumption with parameters obtained by solving the multiple experiment parameter estimation problem. **Figure S9.** Growth rate measurements of wild type cells and cells expressing psd module variants. **Table S1.** List of mutated LOV2 domain residues. **Table S2.** Conversion rate constants of psd module variants obtained by parameter estimation. **Table S3.** Starting values for parameter estimation. **Table S4.** Plasmids used in this study. Supplementary references.

## Abbreviations

psd: Photo-sensitive degron
RFP: Red fluorescent protein
FMN: Flavin mononucleotide
LOV: Light oxygen voltage
ODC: Ornithine decarboxylase
LED: Light-emitting diode
ODE: Ordinary differential equation
BVP: Boundary value problem
chx: Cycloheximide
