# Supplementary material for: Photo-sensitive degron variants for tuning protein stability by light
Source: BMC Syst Biol. 2014 Nov 18;8:128. doi: 10.1186/s12918-014-0128-9 (PMC4236813; doi:10.1186/s12918-014-0128-9)
Supplement: Additional file 1: Figure S1 — Growth behavior of yeast cells in blue light compared to darkness. Figure S2. Mutational analysis of psd module stability. Figure S3. Quantification of psd module variant behavior. Figure S4. The impact of the cysteine within the cODC1 degron on psd module variant stability. Figure S5. Equations used for the in silico analysis of psd module variants. Figure S6. Parameter estimation to the experimental data of the psd module variants. Figure S7. Influence of increased kdegLOV and decreased kdark on psd module behavior. Figure S8. Comparison of human parameter assumption with parameters obtained by solving the multiple experiment parameter estimation problem. Figure S9. Growth rate measurements of wild type cells and cells expressing psd module variants. Table S1. List of mutated LOV2 domain residues. Table S2. Conversion rate constants of psd module variants obtained by parameter estimation. Table S3. Starting values for parameter estimation. Table S4. Plasmids used in this study. Supplementary references. [file 12918_2014_128_MOESM1_ESM.pdf]

Supporting information

Figure S1: Growth behavior of yeast cells in blue light compared to darkness.
Figure S2: Mutational analysis of psd module stability.
Figure S3: Quantification of psd module variant behavior.
Figure S4: The impact of the cysteine within the cODC1 degron on psd module variant stability.
Figure S5: Equations used for the *in silico* analysis of psd module variants.
Figure S6: Parameter estimation to the experimental data of the psd module variants.
Figure S7: Influence of increased k_{degLOV} and decreased k_{dark} on psd module behavior.
Figure S8: Comparison of human parameter assumption with parameters obtained by solving the multiple experiment parameter estimation problem.
Figure S9: Growth rate measurements of wild type cells and cells expressing psd module variants.
Table S1: List of mutated LOV2 domain residues.
Table S2: Conversion rate constants of psd module variants obtained by parameter estimation.
Table S3: Starting values for parameter estimation.
Table S4: Plasmids used in this study.
Supplementary references.

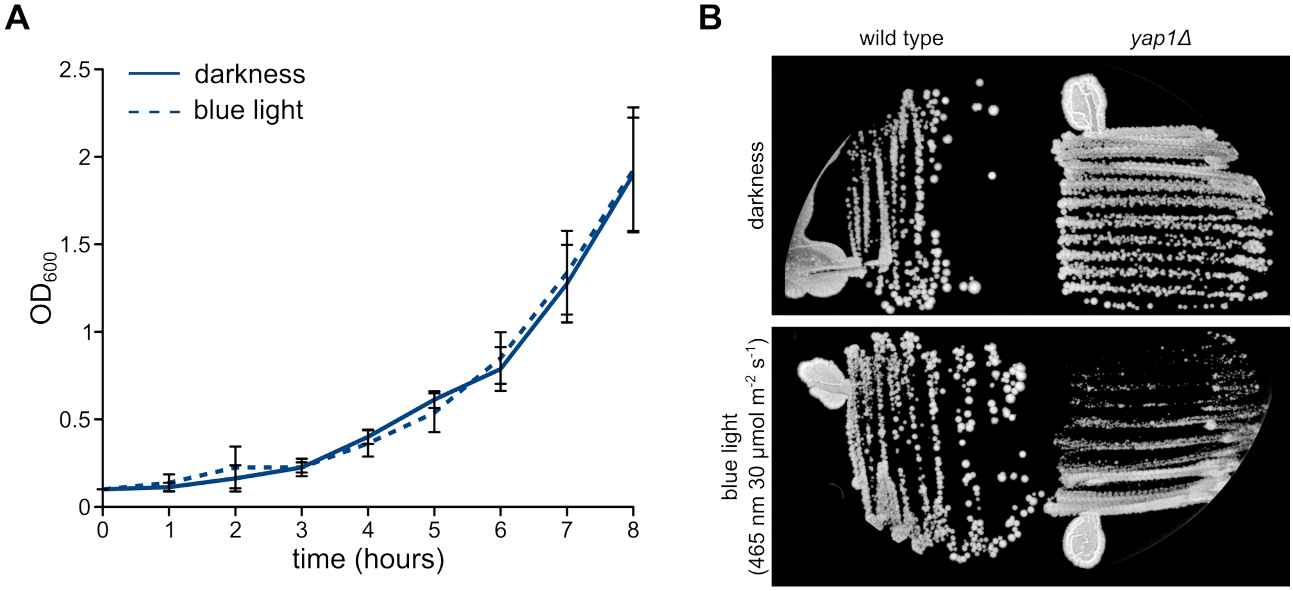

Figure S1
Growth behavior of yeast cells in blue light compared to darkness. A) Growth rate measurements of yeast cells (ESM356-1 carrying plasmid pRS315) in darkness or under blue-light illumination (465 nm 30 µmol m^{-2} s^{-1}) for 8 hours. The optical density at 600 nm (OD_{600}) of the cultures was measured at the indicated time points (error bars: standard deviation, n=4). B) A *YAP1* deletion strain is growing slower when illuminated with blue light. Wild type and *YAP1* deletion cells were streaked on YPD plates and incubated in darkness or under constant blue-light illumination (465 nm 30 µmol m^{-2} s^{-1}) for two days at room temperature.

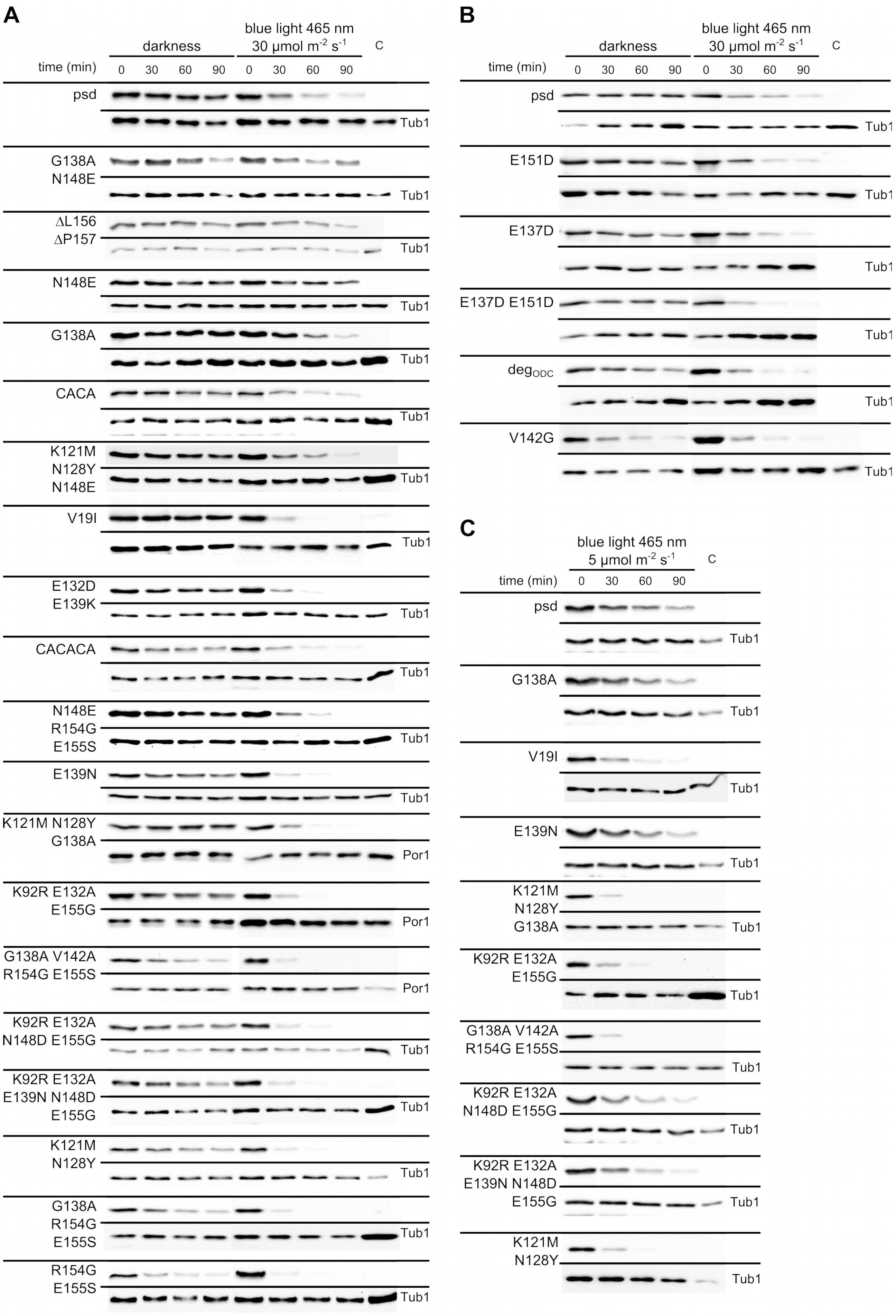
A
darkness
blue light 465 nm
30 µmol m-2 s-1
C
time (min)
0 30 60 90 0 30 60 90
psd
Tub1
G138A N148E
ΔL156 ΔP157
N148E
G138A
CACA
K121M N128Y N148E
V19I
E132D E139K
CACACA
N148E R154G E155S
E139N
K121M N128Y G138A
Por1
K92R E132A E155G
G138A V142A R154G E155S
K92R E132A N148D E155G
K92R E132A E139N N148D E155G
K121M N128Y
G138A R154G E155S
R154G E155S
B
E151D
E137D
E137D E151D
degODC
V142G
C
blue light 465 nm
5 µmol m-2 s-1

Figure S2

Mutational analysis of psd module stability. **A**) Yeast cells expressing P_{ADH1}-*RFP-psd* (plasmid based) or one of the variants (as indicated) were grown in liquid medium in darkness. After taking the first sample (t=0 hours), the translation inhibitor cycloheximide (chx) was added; cells were kept in darkness or were illuminated with blue light (LED lamp, 465 nm, 30 μmol m^{-2} s^{-1}) for the rest of the experiment. Samples were taken at the indicated time points and subjected to alkaline lysis followed by immunoblotting. Antibodies against tRFP and Tub1 or Por1 (loading controls) were used for detection (negative control: C). **B**) Conditions as in **A**. Derivatives of plasmid pDS112 (P_{ADH1}-*GFP-3myc-psd)* were analyzed. Immunoblotting was performed with antibodies against myc and Tub1. **C**) Stability of the psd module and its variants exposed to blue light with an intensity of 5 μmol m^{-2} s^{-1}. Other conditions as in **A**.

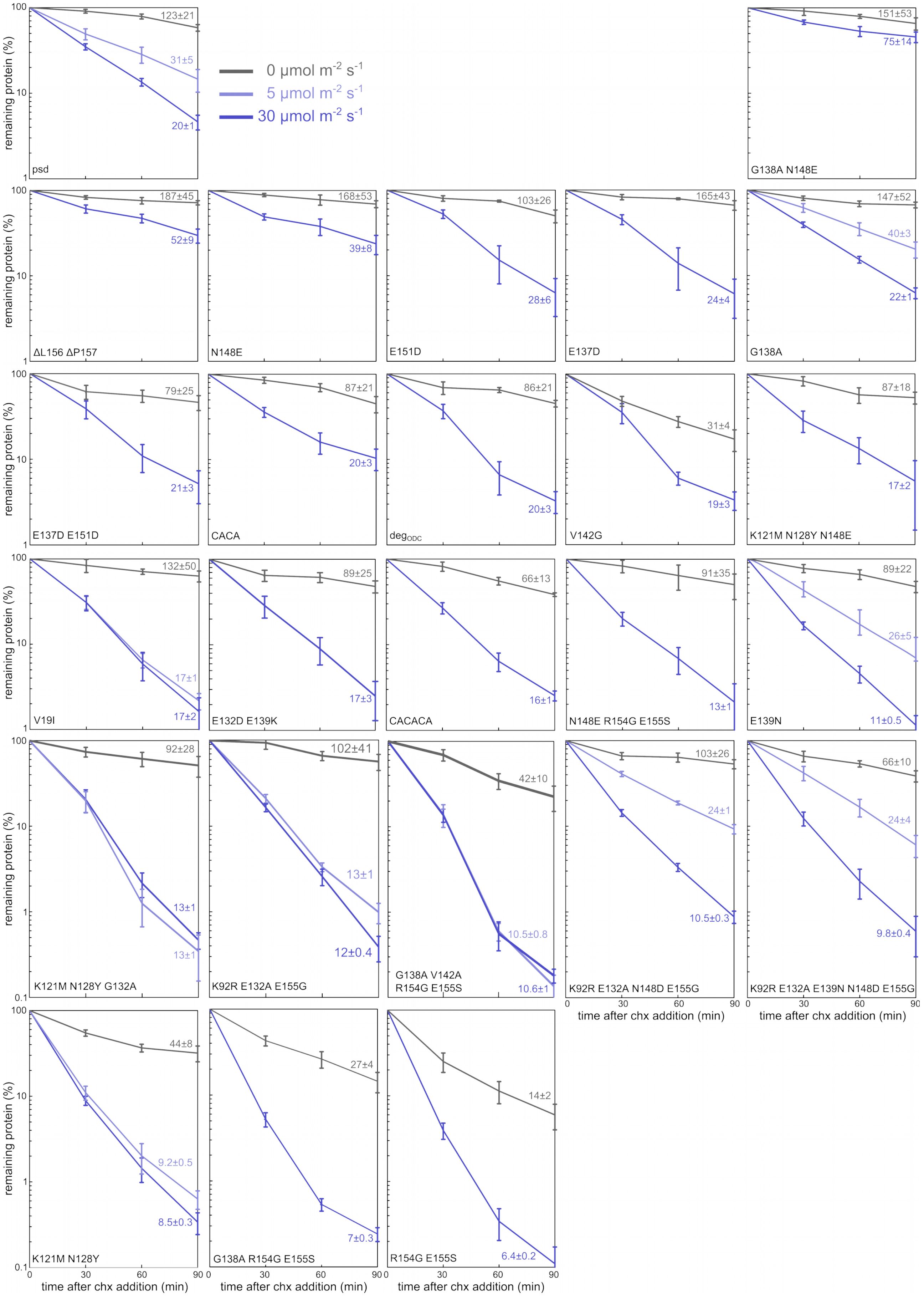

0 µmol m-2 s-1
5 µmol m-2 s-1
30 µmol m-2 s-1
remaining protein (%)
time after chx addition (min)
psd
123±21
31±5
20±1
G138A N148E
151±53
75±14
ΔL156 ΔP157
187±45
52±9
N148E
168±53
39±8
E151D
103±26
28±6
E137D
165±43
24±4
G138A
147±52
40±3
22±1
E137D E151D
79±25
21±3
CACA
87±21
20±3
degODC
86±21
20±3
V142G
31±4
19±3
K121M N128Y N148E
87±18
17±2
V19I
132±50
17±1
17±2
E132D E139K
89±25
17±3
CACACA
66±13
16±1
N148E R154G E155S
91±35
13±1
E139N
89±22
26±5
11±0.5
K121M N128Y G132A
92±28
13±1
13±1
K92R E132A E155G
102±41
13±1
12±0.4
G138A V142A
R154G E155S
42±10
10.5±0.8
10.6±1
K92R E132A N148D E155G
103±26
24±1
10.5±0.3
K92R E132A E139N N148D E155G
66±10
24±4
9.8±0.4
K121M N128Y
44±8
9.2±0.5
8.5±0.3
G138A R154G E155S
27±4
7±0.3
R154G E155S
14±2
6.4±0.2

Figure S3

Quantification of psd-module variant behavior. Curves are the means of protein amounts obtained from at least four independent measurements (error bars: s.e.m.; representative immunoblots are shown in **Figure S2 A, B,** and **C**). The half-lives (in minutes±standard error) for each condition are shown next to each curve.

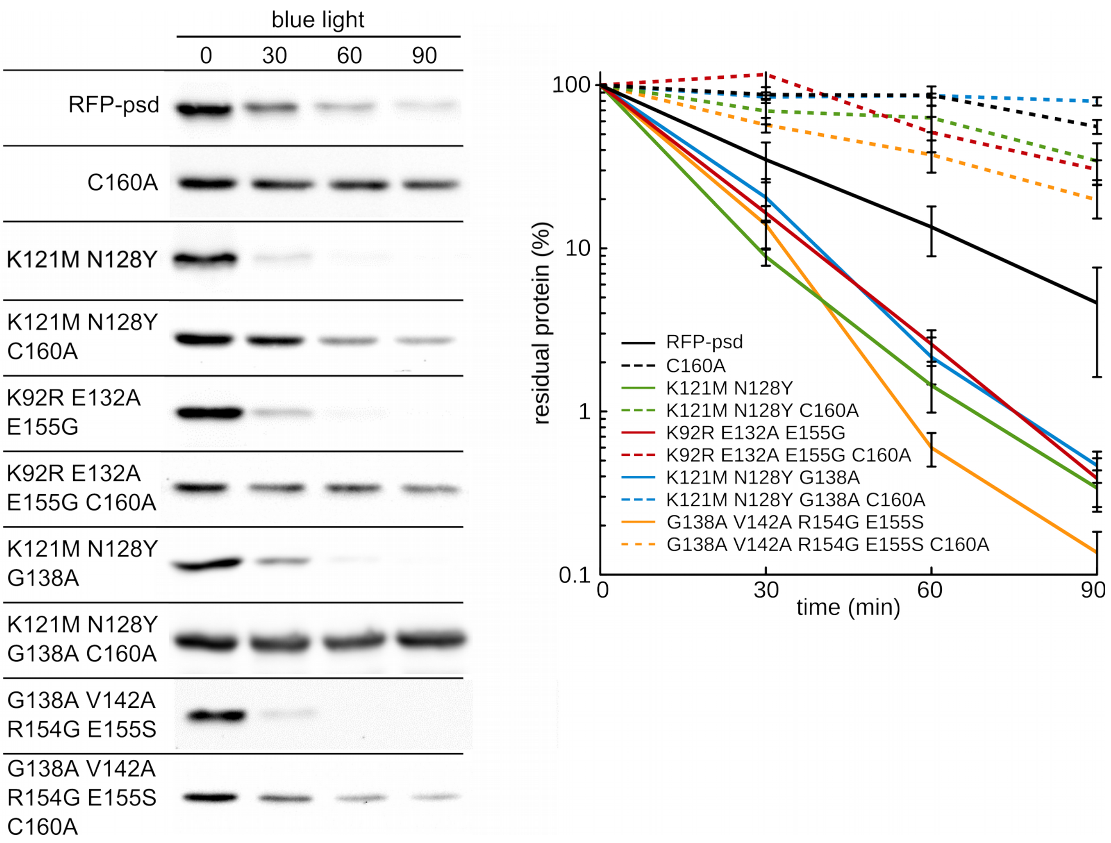

Figure S4

The impact of the cysteine within the cODC1 degron on psd module variant stability. The cysteine of cODC1 is the main determinant of psd module variant degradation in all tested cases (wild type construct, K121M N128Y, K92R E132A E155G, K121M N128Y G138A, and G138A V142A R154G E155S). The variants were expressed in yeast cells (plasmid based) and subjected to cycloheximide chases (as described for Figure S2A) under blue-light illumination (30 µmol m^{-2} s^{-1}). Left side: representative immunoblots; right side: quantification of psd module variant behavior. Four independent measurements were performed for each variant, (error bars: s.e.m.).

A

$$\frac{d[PSD_{dark}]}{dt} = +k_{pp1\_translation\_rate} \cdot [pp1\_mRNA] + k_{dark} \cdot [PSD_{lit}] - k_{hv} \cdot [PSD_{dark}] - k_{degENDO} \cdot [PSD_{dark}] - k_{leak} \cdot [PSD_{dark}]$$

$$\frac{d[PSD_{lit}]}{dt} = -k_{dark} \cdot [PSD_{lit}] + k_{hv} \cdot [PSD_{dark}] - k_{degENDO} \cdot [PSD_{lit}] - k_{degLOV} \cdot [PSD_{lit}] + k_{leak} \cdot [PSD_{dark}]$$

$$\frac{d[pp1\_mRNA]}{dt} = +[psd] - k_{pp1\_translation\_rate} \cdot [pp1\_mRNA]$$

B

$$\frac{d[PSD_{dark}]}{dt} = +k_{dark} \cdot [PSD_{lit}] - k_{hv} \cdot [PSD_{dark}] - k_{degENDO} \cdot [PSD_{dark}] - k_{leak} \cdot [PSD_{dark}]$$

$$\frac{d[PSD_{lit}]}{dt} = -k_{dark} \cdot [PSD_{lit}] + k_{hv} \cdot [PSD_{dark}] - k_{degENDO} \cdot [PSD_{lit}] - k_{degLOV} \cdot [PSD_{lit}] + k_{leak} \cdot [PSD_{dark}]$$

Figure S5

Equations used for the *in silico* analysis of psd module variants. Values for the conversion rate constants k_{dark}, $k_{degENDO}$, k_{degLOV}, k_{leak} are given in Table 2; other values can be found in the description of the simulations in the methods section. **A**) Equations used to simulate reaction of psd module variants towards light. **B**) Equations used for simulation of cycloheximide chase analysis and parameter estimations.

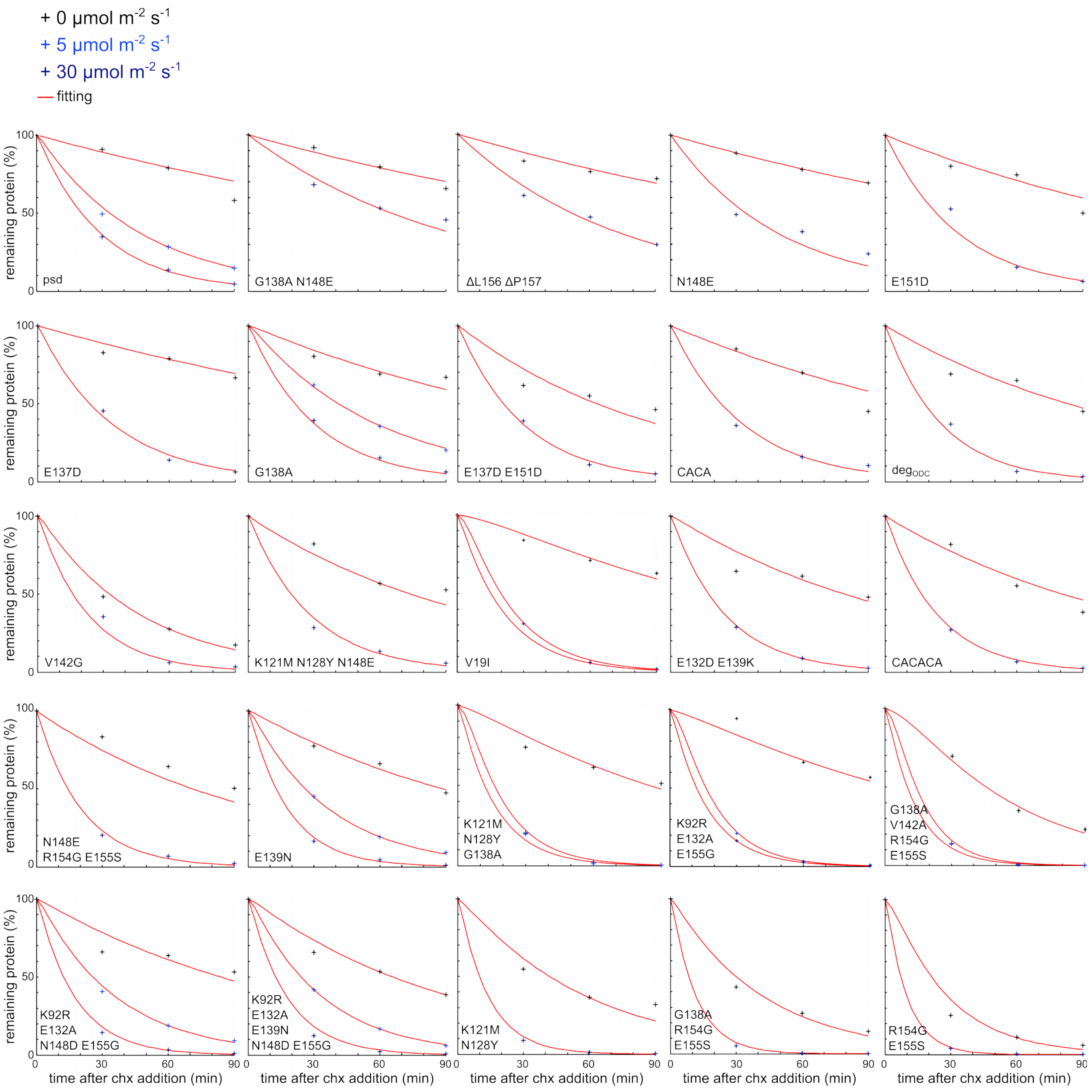

Figure S6

Parameter estimation to the experimental data of the psd module variants. The conversion constants k_{dark}, k_{leak}, k_{degLOV} and $k_{degENDO}$ were adapted to reproduce the experimental data. For the psd module, k_{dark} and k_{leak} were taken from the literature and k_{degLOV} as well as $k_{degENDO}$ were adjusted. Parameter estimation using the 5 µmol m^{-2} s^{-1} data required to adjust the $k_{h\nu}$ value slightly for the psd module.

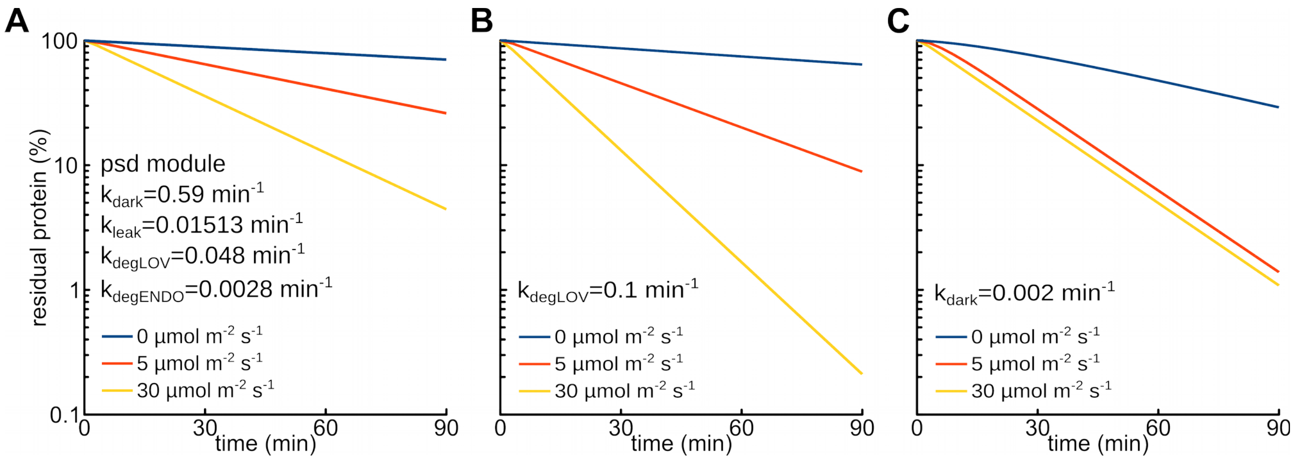

Figure S7

Influence of increased k_{degLOV} and decreased k_{dark} on psd module behavior. **A**) Simulation of the wild type psd module using the parameters obtained by parameter estimation. **B**) Simulation with increased k_{degLOV}, other parameters as in A). **C**) Simulations with decreased k_{dark}, other parameters as in A). The simulations demonstrate the impact of the parameters. Qualitatively, the graph with increased k_{degLOV} is similar to the results obtained by cycloheximide chases for the variants K92R E132A N148D E155G or K92R E132A N148D E155G, whereas the graph with decreased k_{dark} resembles the variants V19I, K121M N128Y, K121M N128Y G138A, K92R E132A E155G or G138A V142A R154G E155S (see Figure S2).

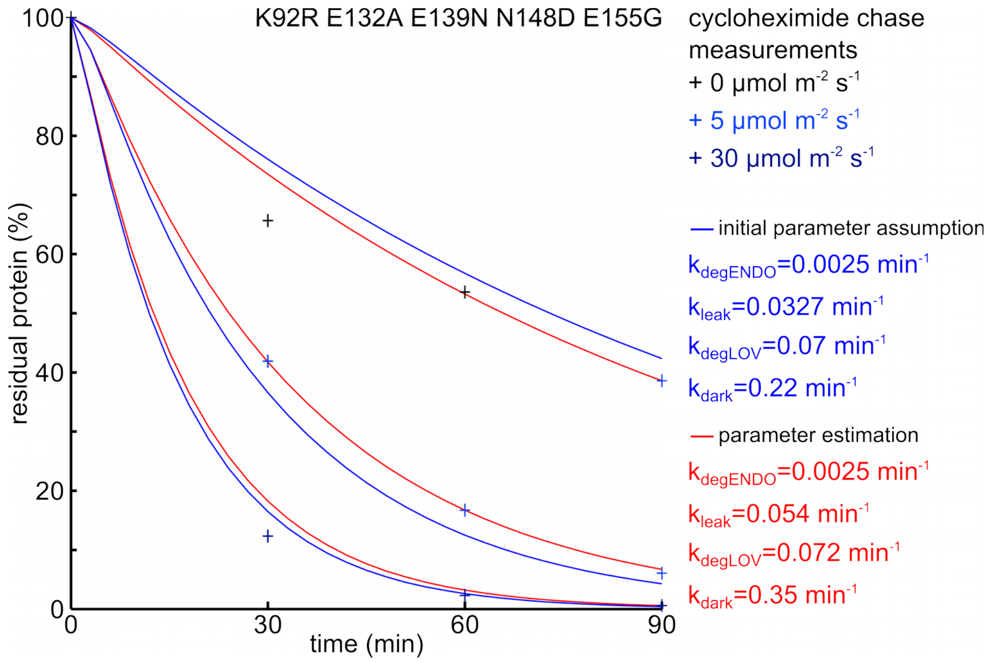

Figure S8

Mathematical-based parameter estimation has higher accuracy than human parameter assumption. Comparison between parameters derived by TinkerCell cycloheximide chase simulations (blue curve) and parameter estimation (red curve) for the psd module variant K92R E132A E139N N148D E155G.

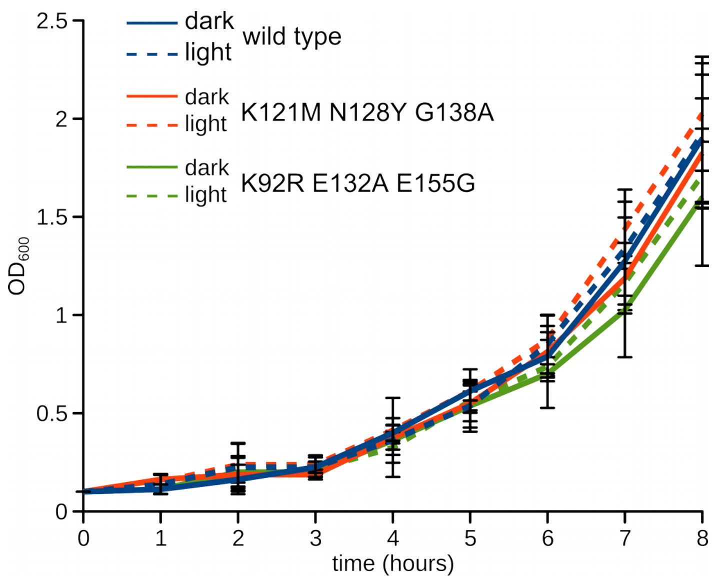

Figure S9

Growth rate measurements of wild type cells and cells expressing psd module variants. Yeast cells (ESM356-1) carrying either an empty plasmid (pRS315), the psd module variant K121M N128Y G138A (pDS142) or K92R E132A E155G (pDS143) were grown in darkness or under blue-light illumination (465 nm 30 µmol m^{-2} s^{-1}) for 8 hours. The optical density at 600 nm (OD_{600}) of the cultures was measured at the indicated time points (error bars: standard deviation, n=4).

Table S1: List of mutated LOV2 domain residues

Name	Residue in full length phototropin1 of *Arabidopsis thaliana*
Wild type psd module	M460 to P616-MSCAQESITSLYKKAGSENLYFQ (cODC1)
V19I	V478I
K92R E132A E155G	K551R E591A E614G
K92R E132A N148D E155G	K551R E591A N607D E614G
K92R E132A E139N N148D E155G	K551R E591A E598N N607D E614G
K121M N128Y	K580M N587Y
K121M N128Y G138A	K580M N587Y G597A
K121M N128Y N148E	K580M N587Y G597A N607E
E132D E139K	E591D E598K
E137D	E596D
E137D E151D	E596D E610D
G138A	G597A
G138A V142A R154G E155S	G597A V601A R613G E614S
G138A N148E	G597A N607E
G138A R154G E155S	G597A R613G E614S
E139N	E598N
V142G	V601A
N148E	N607E
N148E R154G E155S	N607E R613G E614S
E151D	E610D
R154G E155S	R613G E614S
ΔL156 ΔP157	ΔL615 ΔP616
deg_{ODC}	M460 to P616-MSCAQESGMDRHPAACASARINV (murine cODC)
CACA	M460 to P616-MSCACAQESITSLYKKAGSENLYFQ (cODC1-CACA)
CACACA	M460 to P616-MSCACACAQESITSLYKKAGSENLYFQ (cODC1-CACACA)

Table S2: Conversion rate constants of psd module variants obtained by parameter estimation.

Variant name	$k_{dark} \cdot 10^{-2}$ (min^{-1})	$k_{leak} \cdot 10^{-2}$ (min^{-1})	$k_{degLOV} \cdot 10^{-2}$ (min^{-1})	$k_{degENDO} \cdot 10^{-2}$ (min^{-1})
wild type psd module	59 (lit.)	1.513 (lit.)	4.8±0.6	0.28±0.08
V19I	0.7±0.5	0.7±0.1	4.7±0.1	0.1±0.01
K92R E132A E155G	0.4±0.6	0.5±0.1	5.9±0.2	0.3±0.01
K92R E132A N148D E155G	41.2±10.1	4.1±1.9	7.6±0.7	0.25±0.04
K92R E132A E139N N148D E155G	35.2±2.9	5.4±1.2	7.2±0.2	0.25±0.05
K121M N128Y	6.6±8.9	1.5±1.2	6.7±0.6	1.1±0.02
K121M N128Y G138A	0.9±3.7	0.6±0.3	6.0±0.8	0.35±0.01
K121M N128Y N148E	132.5±40.1	0.19±0.16	5.6±0.4	0.93±0.07
E132D E139K	57.8±18.5	8.0±2.4	5.7±0.4	0.25±0.02
E137D	124.7±26.6	3.8±1.0	5.5±0.4	0.25±0.02
E137D E151D	86.7±97.8	15.1±9.9	5.0±2.2	0.4±0.05
G138A	39.1±9.2	3.9±2.1	4.1±0.4	0.25±0.03
G138A V142A R154G E155S	0.5±1.4	2.0±0.3	7.4±0.5	0.2±0.05
G138A N148E	837.3±178.5	22.3±13.2	5.6±0.5	0.25±0.02
G138A R154G E155S	33.7±14.5	6.9±4.1	10.1±0	1±0.03
E139N	37.9±8.1	3.7±0.6	7.1±0.7	0.25±0.05
V142G	46.1±30.3	27.0±13.3	5.5±0.7	0.25±0.03
N148E	271.0±66.4	8.1±5.1	5.6±0.6	0.25±0.03
N148E R154G E155S	189.3±23.8	0.05±0.7	10.4±0	0.97±0.07
E151D	98.6±3.0	3.9±99.4	4.7±2.1	0.4±0.04
R154G E155S	6.8±7.4	5.8±2.7	10.1±1.1	1±0.15
ΔL156 ΔP157	284.0±263.2	10.4±6.5	3.4±2.1	0.3±0.03
deg_{ODC}	59 (lit.)	1.513 (lit.)	4.8±0.4	0.72±0.07
CACA	59 (lit.)	1.513 (lit.)	3.8±0.4	0.52±0.04
CACACA	59 (lit.)	1.513 (lit.)	5.3±0.7	0.74±0.1

Lit. indicates values calculated from literature that were kept fixed during the parameter estimations.

Table S3: Starting values for parameter estimation

Variant name	k_{dark} ·10^{-2} (min^{-1})	k_{leak} ·10^{-2} (min^{-1})	k_{degLOV} ·10^{-2} (min^{-1})	$k_{degENDO}$ ·10^{-2} (min^{-1})
Wild type psd module	59 (fixed)	1.513 (fixed)	5.64 (±10 %)	0.25 (±10 %)
V19I	1 (±100 %)	0.7	4.76 (±10 %)	0.1 (±10 %)
K92R E132A E155G	1	1	6 (±10 %)	0.3 (±10 %)
K92R E132A N148D E155G	30	3	7	0.25 (±10 %)
K92R E132A E139N N148D E155G	220	3.27	7 (±10 %)	0.25 (±10 %)
K121M N128Y	10 (±50 %)	0.5	5.64 (±10 %)	1 (±10 %)
K121M N128Y G138A	3	1	6.74 (±40 %)	0.3 (±10 %)
K121M N128Y N148E	200	2 (±100 %)	5.64 (±10 %)	1 (±10 %)
E132D E139K	55	65	5.64 (±10 %)	0.25 (±10 %)
E137D	70	1.513	5.5 (±10 %)	0.25 (±10 %)
E137D E151D	80 (±100 %)	6	5.5	0.4 (±10 %)
G138A	100	1.92	5.64 (±10 %)	0.25 (±10 %)
G138A V142A R154G E155S	3 (±40 %)	3	9	0.2 (±10 %)
G138A N148E	560	0.5	5.64 (±10 %)	0.25 (±10 %)
G138A R154G E155S	5	2.5	8 (±10 %)	1 (±10 %)
E139N	20	1.65	5.64 (±10 %)	0.25 (±10 %)
V142G	7	40	5.5 (±10 %)	0.25 (±10 %)
N148E	300	0.5	5.64 (±10 %)	0.25 (±10 %)
N148E R154G E155S	150	0.2 (±100 %)	8 (±10 %)	1 (±10 %)
E151D	100 (±100 %)	1.513	5.5	0.4 (±10 %)
R154G E155S	5	5	8	1 (±10 %)
ΔL156 ΔP157	280	0.2	4	0.3 (±10 %)
deg_{ODC}	59 (fixed)	1.513 (fixed)	5.5	0.65
CACA	59 (fixed)	1.513 (fixed)	5.64	0.5 (±10 %)
CACACA	59 (fixed)	1.513 (fixed)	7.5	0.5 (±10 %)

The starting values for parameter estimation were obtained from TinkerCell simulations of cycloheximide chases. For each variant, parameters were chosen that approximated the experimental data. These parameters were then used for the parameter estimation. Values in brackets indicate the variability during parameter estimation, values lacking restriction were freely estimated.

Table S4: plasmids used in this study

Name	Genotype	Source
pRS315	*LEU2 ARS209/CEN2 ori bla*	[1]
pCT334	pRS315 P_{ADH1}-*tagRFP-AtLOV2-cODC1*	[2]
pDS112	pRS315 P_{ADH1}-*GFP-3myc-AtLOV2-cODC1*	This study
pDS133	pRS315 P_{ADH1}-*tagRFP-AtLOV2-cODC1*^{V19I}	This study
pDS143	pRS315 P_{ADH1}-*tagRFP-AtLOV2-cODC1*$^{K92R\ E132A\ E155G}$	This study
pSU17	pRS315 P_{ADH1}-*tagRFP-AtLOV2-cODC1*$^{K92R\ E132A\ N148D\ E155G}$	This study
pSU21	pRS315 P_{ADH1}-*tagRFP-AtLOV2-cODC1*$^{K92R\ E132A\ E139N\ N148D\ E155G}$	This study
pSU23	pRS315 P_{ADH1}-*tagRFP-AtLOV2-cODC1*$^{K121M\ N128Y}$	This study
pDS142	pRS315 P_{ADH1}-*tagRFP-AtLOV2-cODC1*$^{K121M\ N128Y\ G138A}$	This study
pSU16	pRS315 P_{ADH1}-*tagRFP-AtLOV2-cODC1*$^{K121M\ N128Y\ N148E}$	This study
pSU19	pRS315 P_{ADH1}-*tagRFP-AtLOV2-cODC1*$^{E132D\ E139K}$	This study
pDS124	pRS315 P_{ADH1}-*GFP-3myc-AtLOV2-cODC1*^{E137D}	This study
pDS128	pRS315 P_{ADH1}-*GFP-3myc-AtLOV2-cODC1*$^{E137D\ E151D}$	This study
pDTS1	pRS315 P_{ADH1}-*tagRFP-AtLOV2-cODC1*^{G138A}	This study
pDS144	pRS315 P_{ADH1}-*tagRFP-AtLOV2-cODC1*$^{G138A\ V142A\ R154G\ E155S}$	This study
pCT341	pRS315 P_{ADH1}-*tagRFP-AtLOV2-cODC1*$^{G138A\ N148E}$	This study
pDS140	pRS315 P_{ADH1}-*tagRFP-AtLOV2-cODC1*$^{G138A\ R154G\ E155S}$	This study
pSU18	pRS315 P_{ADH1}-*tagRFP-AtLOV2-cODC1*^{E139N}	This study
pDS139	pRS315 P_{ADH1}-*GFP-3myc-AtLOV2-cODC1*^{V142G}	This study
pCT340	pRS315 P_{ADH1}-*tagRFP-AtLOV2-cODC1*^{N148E}	This study
pSU20	pRS315 P_{ADH1}-*tagRFP-AtLOV2-cODC1*$^{N148E\ R154G\ E155S}$	This study
pDS125	pRS315 P_{ADH1}-*GFP-3myc-AtLOV2-cODC1*^{E151D}	This study
pDTS12	pRS315 P_{ADH1}-*tagRFP-AtLOV2-cODC1*$^{R154G\ E155S}$	This study
pDTS4	pRS315 P_{ADH1}-*tagRFP-AtLOV2-cODC1*$^{\Delta L156\ \Delta P157}$	This study
pDS132	pRS315 P_{ADH1}-*GFP-3myc-AtLOV2-deg*$_{ODC}$	This study
pDS109	pRS315 P_{ADH1}-*tagRFP-AtLOV2-cODC1*^{CACA}	This study
pDS110	pRS315 P_{ADH1}-*tagRFP-AtLOV2-cODC1*^{CACACA}	This study
pCT337	*tagRFP-AtLOV2-cODC1::kanMX ori bla*	[2]
pDS91	*tagRFP-AtLOV2-cODC1*^{N148E}*::kanMX ori bla*	This study
pDS90	pRS315 P_{TDH3}-*tagRFP-AtLOV2-cODC1*	This study
pSU9	pRS315 P_{TDH3}-*tagRFP-AtLOV2-cODC1*$^{K121M\ N128Y\ N148E}$	This study
pSU10	pRS315 P_{TDH3}-*tagRFP-AtLOV2-cODC1*$^{K92R\ E132A\ N148D\ E155G}$	This study
pDS87	pRS315 P_{ADH1}-*tagRFP-AtLOV2-cODC1*^{C160A}	[2]
pCT344	pRS315 P_{ADH1}-*tagRFP-AtLOV2-cODC1*$^{K121M\ N128Y\ G138A\ C160A}$	This study
pCT345	pRS315 P_{ADH1}-*tagRFP-AtLOV2-cODC1*$^{K121M\ N128Y\ C160A}$	This study

pCT346	pRS315 P_{ADH1}-*tagRFP-AtLOV2-cODC1*$^{K92R\ E132A\ E155GC160A}$	This study
pCT347	pRS315 P_{ADH1}-*tagRFP-AtLOV2-cODC1*$^{G138A\ V142A\ R154G\ E155S\ C160A}$	This study

Supplementary references

1. Sikorski RS, Hieter P: **A system of shuttle vectors and yeast host strains designed for efficient manipulation of DNA in Saccharomyces cerevisiae.** *Genetics* 1989, **122:**19-27.
2. Renicke C, Schuster D, Usherenko S, Essen LO, Taxis C: **A LOV2 domain-based optogenetic tool to control protein degradation and cellular function.** *Chem Biol* 2013, **20:**619-626.
